# Diagnostic efficacy of contrast-enhanced gastric ultrasonography in staging gastric cancer: a meta-analysis

**DOI:** 10.1186/s12885-024-12210-z

**Published:** 2024-04-05

**Authors:** Yuan Zhong, Yan-Yan Xiao, Jie-Yi Ye, Guo-Liang Jian, Wei-Jun Huang

**Affiliations:** Department of Medical Ultrasound, The First People’s Hospital of Foshan, No. 81 Lingnan Avenue North, Foshan, 528010 China

**Keywords:** Gastric ultrasonography, Contrast agent, Gastric cancer, T-stage, Meta-analysis

## Abstract

**Background:**

As comprehensive surgical management for gastric cancer becomes increasingly specialized and standardized, the precise differentiation between ≤T1 and ≥T2 gastric cancer before endoscopic intervention holds paramount clinical significance.

**Objective:**

To evaluate the diagnostic efficacy of contrast-enhanced gastric ultrasonography in differentiating ≤T1 and ≥T2 gastric cancer.

**Methods:**

PubMed, Web of Science, and Medline were searched to collect studies published from January 1, 2000 to March 16, 2023 on the efficacy of either double contrast-enhanced gastric ultrasonography (D-CEGUS) or oral contrast-enhanced gastric ultrasonography (O-CEGUS) in determining T-stage in gastric cancer. The articles were selected according to specified inclusion and exclusion criteria, and the quality of the included literature was assessed using the Quality Assessment of Diagnostic Accuracy Studies-2 scale. Meta-analysis was performed using Stata 12 software with data from the 2 × 2 crosslinked tables in the included literature.

**Results:**

In total, 11 papers with 1124 patients were included in the O-CEGUS analysis, which revealed a combined sensitivity of 0.822 (95% confidence interval [CI] = 0.753–0.875), combined specificity of 0.964 (95% CI = 0.925–0.983), and area under the summary receiver operating characteristic (sROC) curve (AUC) of 0.92 (95% CI = 0.89–0.94). In addition, five studies involving 536 patients were included in the D-CEGUS analysis, which gave a combined sensitivity of 0.733 (95% CI = 0.550–0.860), combined specificity of 0.982 (95% CI = 0.936–0.995), and AUC of 0.93 (95% CI = 0.91–0.95). According to the I^2^ and P values ​​of the forest plot, there was obvious heterogeneity in the combined specificities of the included papers. Therefore, the two studies with the lowest specificities were excluded from the O-CEGUS and D-CEGUS analyses, which eliminated the heterogeneity among the remaining literature. Consequently, the combined sensitivity and specificity of the remaining studies were 0.794 (95% CI = 0.710–0.859) and 0.976 (95% CI = 0.962–0.985), respectively, for the O-CEDUS studies and 0.765 (95% CI = 0.543–0.899) and 0.986 (95% CI = 0.967–0.994), respectively, for the D-CEGUS studies. The AUCs were 0.98 and 0.99 for O-CEGUS and D-CEGUS studies, respectively.

**Conclusion:**

Both O-CEGUS and D-CEGUS can differentiate ≤T1 gastric cancer from ≥T2 gastric cancer, thus assisting the formulation of clinical treatment strategies for patients with very early gastric cancer. Given its simplicity and cost-effectiveness, O-CEGUS is often favored as a staging method for gastric cancer prior to endoscopic intervention.

## Introduction

Gastric cancer has been listed as the malignancy with the fifth-highest incidence rate and the fourth-highest mortality rate worldwide, according to the Global Cancer Statistics 2020 report [1]. Because of the complexity of gastric cancer management, adequate preoperative staging is prerequisite for rationalizing patient treatment options with the specialization and standardization of comprehensive surgical management for gastric cancer.

Surgical resection and partial combination chemotherapy have been clinically preferred when patients have progressive gastric cancer (i.e., stage ≥T2 gastric cancer) [2]. However, studies previously demonstrated that endoscopic submucosal dissection (ESD) is a feasible option for treating early gastric cancer (i.e., stage ≤T1 gastric cancer) without lymph node metastasis, provided the indications are met. Because ESD is a less invasive procedure with comparable near- and long-term efficacy as traditional surgical procedures [3], it can potentially change the future paradigm of gastric cancer management. Therefore, accurately differentiating stage ≤T1 gastric cancer from stage ≥T2 gastric cancer before endoscopic intervention carries clinical significance.

Currently, the primary methods for diagnosing gastric lesions are gastroscopy, endoscopic ultrasonography (EUS), computed tomography (CT), double contrast-enhanced gastric ultrasonography (D-CEGUS), and oral contrast-enhanced gastric ultrasonography (O-CEGUS) [4–7]. Gastroscopy allows direct visualization and biopsy of gastric cancer. However, it does not determine the depth of tumor infiltration (i.e., T-staging). EUS is considered mandatory preoperatively for patients recommended for ESD [2]. However, it is invasive, it is susceptible to inflammation and the probe angle, and it is demanding on the operator. CT utilizes ionizing radiation, which is harmful to patients, and it has limitations for observing the submucosal hypodense zone (stage ≤T1).

CEGUS is a widely accessible and radiation-free imaging technique that can display the hierarchical structure of the gastric wall and lesions, and it is gradually gaining clinical acceptance as an essential tool for the mass screening of gastric cancer [5–8]. Despite many clinical studies on the feasibility of contrast-enhanced gastric ultrasonography in the preoperative evaluation of gastric cancer, its applicability in clinically staging gastric cancer remains unclear. Therefore, this meta-analysis examined the diagnostic utility of O-CEGUS/D-CEGUS in differentiating ≤T1 and ≥T2 gastric cancer for selecting suitable treatments.

## Methods

### Search strategy and selection criteria

We searched PubMed (National Center for Biotechnology Information, Bethesda, MD, USA), Web of Science (Thomson Reuters, Toronto, Canada), and Medline (using OvidSP, US National Library of Medicine, Bethesda, MD, USA) to identify studies published from January 1, 2000 to March 16, 2023 on the use of either O-CEGUS or D-CEGUS in differentiating stage ≤T1 and stage ≥T2 gastric cancer (based on the American Joint Committee on Cancer [AJCC]/International Union against Cancer [UICC] TNM staging system). The medical subject headings used for the search were “((gastr* OR stomach) AND (carcinoma OR cancer OR tumor OR neoplas* OR disease) AND ultraso*).” Additionally, “(NOT endoscop*)” was used to reduce the number of results. In addition, all published meta-analyses on similar topics were reviewed.

This study included both English or Chinese reports of clinical trials and cohort studies evaluating the diagnostic efficacy of either O-CEGUS or D-CEGUS in staging gastric cancer or determining the depth of infiltration. Eligible studies encompassed patients with well-defined postoperative pathological findings (gold standard), particularly pertaining to the T-stage according to the AJCC/UICC TNM staging system category (T1, T2, T3, and T4). Moreover, patients were required to undergo a comprehensive preoperative examination using O-CEGUS or D-CEGUS. In addition, the included studies provided sufficient information to construct a 2 × 2 column table to categorize the diagnostic accuracy of CEGUS for stage ≤T1 or ≥T2 gastric cancer (true positive, false positive, false negative, and true negative; Table 1).

**Table 1:** Cross-contingency table of D-CEGUS/O-CEGUS in differentiating ≤T1 and ≥T2 gastric cancer

D-CEGUS/O-CEGUS	Gold standard	
	≤T1	T2-T4
≤T1	TP	FP
T2-T4	FN	TN

*D-CEGUS* Double contrast-enhanced gastric ultrasonography, *O-CEGUS* Oral contrast-enhanced gastric ultrasonography, *TP* true positives, *FP* false positives, *FN* false negatives, *TN* true negatives

Furthermore, studies involving animal experiments, reviews, correspondence, case reports, expert opinions, and editorials were excluded. Additionally, to ensure the robustness of the findings, only the largest studies were included in cases of overlap among study populations. Adhering to the guidelines outline in the Preferred Reporting Items for Systematic Reviews, the present investigation aimed to maintain methodological rigor and transparency throughout the review process [9].

### Procedures

After excluding duplicates using EndNote X9, two researchers (ZY, XYY) independently screened the literature and extracted information based on aforementioned selection criteria. Generally, the researchers first read the titles and abstracts to exclude unsuitable studies. Then, the researchers thoroughly read the full texts of the included studies to further eliminate literature with incomplete or inadequate information.

The data that were extracted from the selected literature study included the name of first author of the study, the year of publication, and country (Table 2). Two researchers (ZY and XYY) independently assessed the quality of the included literature using RevMan 5.3 software (Copenhagen: The Nordic Cochrane Center, The Cochrane Collaboration, 2014) and Quality Assessment of Diagnostic Accuracy Studies-2 (University, Bristol, UK) [10]. Two investigators discussed and resolved any disagreement during the selection process, and a third investigator (YJY) was involved if a consensus was not reached between the two investigators.

**Table 2:** The basic information of the included studies

**Author**	**Year**	**Country**	**PL (C/E)**	**Design(R/P)**	**Blind**	**Number**	**Gender(F/M)**	**Average age ±SD (years)**	**TNM edition**	**Agents**	**Machine**	**Scheme(D/O)**	**≤T1 VS ≥T2**			
													**TP**	**FP**	**FN**	**TN**
Cheng-long Wang	2009	China	C	R	DB	59	24/38	56.0 ± 11.4	UICC 6th	Contrast medium	Acuson Sequoia 512	O	2	1	1	55
												D	2	0	1	56
Jian Cui	2010	China	C	R	DB	59	N	N	UICC 6th	water	Acuson Sequoia 512	O	2	15	1	41
												D	2	6	1	50
Kengo Sato^a^	2017	Japan	E	R	DB	N	N	N	The Japanese version^c^	Water	APLIO XG/SSA-790A, APLIO 500 TUS一A500	O	89	13	11	79
Shuxiang Zhang	2018	China	E	R	DB	72	27/45	46.9±11.3	UICC 8th	Contrast medium	GE Voluson730	O	14	2	6	50
Ping He	2019	China	E	R	DB	54	18/36	61±9.70	AJCC 8th	water	Philips iU22	D	7	0	1	46
Q.Z. Gai	2021	China	E	R	DB	109	41/68	51.37±11.45	AJCC 8th	Contrast medium	Philips iU22	O	20	2	5	82
Zhixiang Gao	2022	China	E	R	DB	46	N	N	N	Water	Philips iU22	O	3	3	0	40
LING-LING Wu	2023	China	E	P	DB	108	29/79	61.7±11.2	AJCC 8th	Contrast medium	GE LOGIQ E9	O	16	3	3	86
Liang Wang	2019	China	E	R	DB	158	52/106	59.5±10.6	N	Contrast medium	Acuson Sequoia 512	D	20	3	12	123
Junling Wang	2021	China	E	R	DB	206	95/111	59.7±11.3	AJCC 8th	Contrast medium	Acuson Sequoia 512	D	10	3	1	192
Tao Yu	2015	China	E	R	DB	40	13/27	49(25~73)^b^	AJCC 7th	Contrast medium	GE Logiq E9	O	5	1	0	34
Zhijun Liu	2015	China	E	P	DB	264	N	N	N	Contrast medium	Toshiba Aplio 400	O	32	4	4	224
Sheng-Ri Liao	2004	China	E	R	DB	125	47/78	N	AJCC 6th	Water	Toshiba 6000, Aloka 2000, DU-6	O	5	2	4	114
Sainan Wang	2022	China	E	R	DB	50	28/22	25.5±25	N	Contrast medium	MINDRAY Resona 7T	O	8	0	4	38

^a^This study included 190 patients with a total of 200 lesions, of which 8 lesions were unclear

^b^Median age (range)

^c^The TNM criteria of the Japanese Classification of Gastric Carcinoma

*PL* publication language, *SD* Standard deviation, *C* Chinese, *E* English, *P* Prospective study, *R* Retrospective study, *DB* Double blind design, *N* Not unclear, *TP* True positive, *FP* False positive, *FN* False negative, *TN* True negative, *UICC* Union for International Cancer Control, *AJCC* American Joint Committee on Cancer, *D* Double contrast enhanced gastric ultrasonograp, *O* Oral contrast-enhanced gastric ultrasonography

### Statistical analysis

We presented true-positive, false-negative, false-positive, and true-negative data for the differential diagnosis of gastric cancer at stage ≤T1 or ≥T2 by O-CEGUS and/or D-CEGUS in each study separately to construct 2 × 2 cross-tabulation tables, which were used to calculate the sensitivity and specificity of gastric filling ultrasonography for differentiating stage ≤T1 gastric cancer from stage ≥T2 gastric cancer. If both ultrasonographic protocols were analyzed in a single study (O-CEGUS and D-CEGUS), we separately collected data for both procedures in our analyses. In addition, when documenting the accuracy of a study by different readers, we agreeably used the numbers provided by the first reader.

Statistical analyses were performed using Stata 14.0 (Stata, College Station, TX, USA) and MetaDisc 1.4 (Ramón y Cajal Hospital, Madrid, Spain) [11]. Data from each independent study were used to calculate the combined sensitivity, specificity, positive likelihood ratio (PLR), negative likelihood ratio (NLR), and diagnostic advantage ratio with their corresponding 95% confidence intervals (CIs). PLR was defined as the occurrence of a positive O-CEGUS/D-CEGUS diagnosis in patients with stage ≤T1 gastric cancer in discriminating stage ≤T1 gastric cancer from stage ≥T2 gastric cancer. NLR was defined as the likelihood of a negative O-CEGUS/D-CEGUS diagnosis in patients with stage ≥T2 gastric cancer in distinguishing stage ≤T1 gastric cancer from stage ≥T2 gastric cancer. The diagnostic odds ratio was defined as the ratio of the likelihood of a positive O-CEGUS/D-CEGUS result in patients with stage ≤T1 gastric cancer to the likelihood of a positive O-CEGUS/D-CEGUS result in patients with stage ≥T2 gastric cancer.

Because O-CEGUS and D-CEGUS are two different examination protocols, forest plots were used to present the combined sensitivity and specificity of O-CEGUS and D-CEGUS for differentiating stage ≤T1 gastric cancer from stage ≥T2 gastric cancer along with summary receiver operating characteristic (sROC) curves. Moreover, κ statistics were used to quantitatively assess the concordance between the two investigators in evaluating the quality of the literature. Heterogeneity in test precision was examined for each forest plot using the index of inconsistency (I^2^) and the Cochran Q statistic [12]. In the analysis, *P* ≥ 0.05 denoted a lack of no significant difference, indicating no heterogeneity among the studies, and the effect sizes could be combined using a fixed-effects model. However, *P* < 0.05 suggested a significant difference, denoting heterogeneity among the studies exists, and the effect sizes could be combined using a random-effects model. Low, moderate, and high heterogeneity were indicated by *I*^2^ ≤25%, 25% < *I*^2^ ≤ 50%, and *I*^2^ > 50%, respectively [13]. Heterogeneity among the studies was assumed when *I*^2^ > 50% and *P* < 0.05, and a random-effects model was used for statistical analysis.

Diagnostic analysis was performed using sROC curves. After plotting the corresponding sROC curves, the area under the curve (AUC) was calculated to determine its diagnostic value. Publication bias analysis was performed using Deek’s test for studies that could provide information on the diagnostic tetragonal table for discriminating stage ≤T1 gastric cancer from stage ≥T2 gastric cancer. Publication bias was considered to exist among the included literature if *P* < 0.05. Finally, sensitivity analysis was used to verify the stability of the model.

### O-CEGUS/D-CEGUS scanning

Before the O-CEGUS examination, all patients fasted for more than 6–8 h. The ultrasonographic scan began with an essential two-dimensional ultrasound examination using an abdominal probe to identify various stomach lesions. Next, the patients were instructed to consume oral contrast or water (500–800 mL) to fill the gastric cavity, followed by scans in the supine and lateral positions. The presence, location, size, echogenic features, morphology, and stomach wall hierarchy of gastric lesions were imaged and recorded [2, 5, 6]. D-CEGUS was performed by pushing 2.4 mL of intravenous contrast (SonoVue®) through the median cubital vein along with O-CEGUS. Afterward, the whole stomach was scanned in the contrast pulse sequencing mode, and all related information was recorded [14].

The T-staging criteria for both D-CEGUS and O-CEGUS relied on the five-layer structure of the gastric wall. T1 denoted tumor invasion confined to the first three layers from the stomach cavity outward, specifically within the mucosa or submucosa. T2 indicated tumor invasion to the fourth layer, extending outward to the muscular propria. T3 represented tumor penetration of the fifth layer, signifying invasion of the serous layer. Lastly, T4 indicated tumor penetration through the serous layer, with possible invasion into adjacent tissues or organs. These defined criteria provide a structured framework for accurately staging gastric cancer based on the depth of tumor invasion, facilitating effective clinical decision-making and treatment planning.

## Results

### Literature retrieval results

Using the search strategy, 685 papers were obtained, of which 419 papers remained after de-duplication using EndNote. After reading the titles and abstracts, 29 papers were chosen for full reading, and 17 did not meet the inclusion criteria or did not provide sufficient data to the complete four-cell table for differentiating stage ≤T1 and stage ≥T2 gastric cancer. Thus, 12 studies [15–26] were finally included. Among the 390 papers excluded from the initial screening, four meta-analyses related to the T-stage or depth of infiltration of gastric cancer diagnosed by either O-CEGUS or D-CEGUS were identified. Of these, two studies were found to provide sufficient data to complete the four-cell table (true positive, false positive, true negative, false negative) for differentiating stage ≤T1 and stage ≥T2 gastric cancer and were included [14, 27]. Altogether, 14 publications were included in this meta-analysis. The basic information of all included articles is presented in Table 2.

**Fig. 1:**
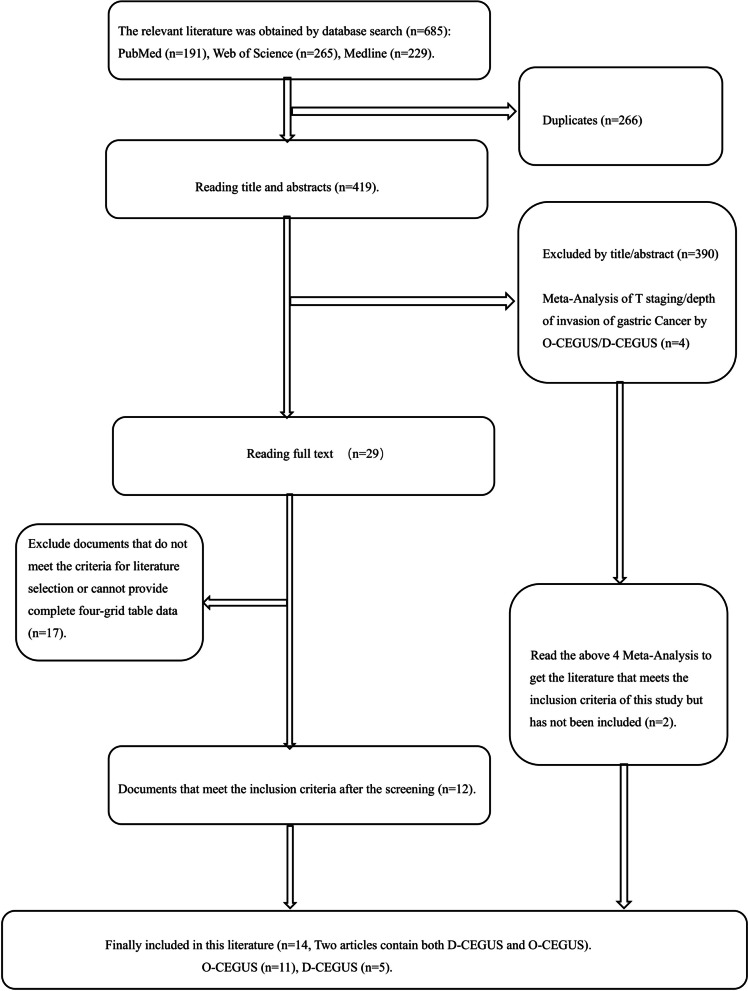
Flow chart of literature screening

**Fig. 2:**
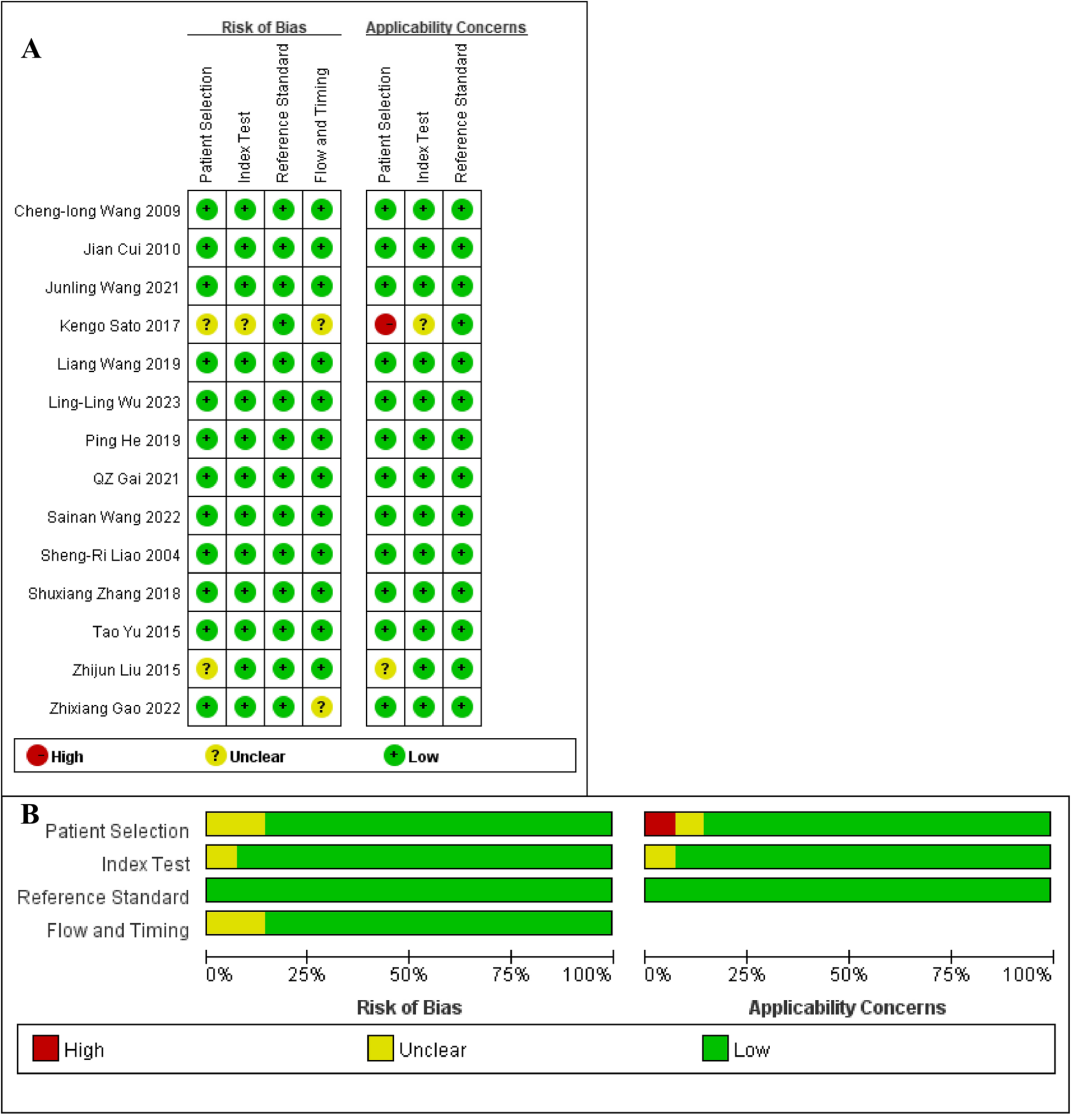
Risk of bias and applicability concerns. **A** Quality evaluation of the included studies (*n* = 14). Red indicates high risk, green indicates low risk, and yellow indicates incomplete information that cannot be assessed. **B** Authors’ judgments about each domain presented as percentages across the included studies

Because O-CEGUS and D-CEGUS are performed using two different operation protocols, we analyzed the two protocols separately. Among the 14 included studies, two investigated the use of both O-CEGUS and D-CEGUS in staging early gastric cancer, and thus, these studies were included in both the O-CEGUS and D-CEGUS analyses. Collectively, a pool of 11 papers, as well as another pool of five papers, were included in the meta-analysis on either O-CEGUS or D-CEGUS in staging early gastric cancer (≤T1 stage gastric cancer). The flow chart of the literature screening is presented in Fig. 1. There was high agreement between the two observers in the assessment of the quality of the literature (κ = 0.900). In summary, 1124 patients in 11 papers on O-CEGUS in staging gastric cancer were included in this meta-analysis, with the study populations ranging from 40 to 264, and 536 patients in five papers on D-CEGUS in gastric cancer staging were included in the meta-analysis, with the study populations ranging 54 to 206. Furthermore, the CEGUS-based diagnosis was confirmed by surgical pathology results. All of the information about the included studies and evaluation of the quality of the literature is presented in Table 2 and Fig. 2.

### Meta-analysis

**Fig 3:**
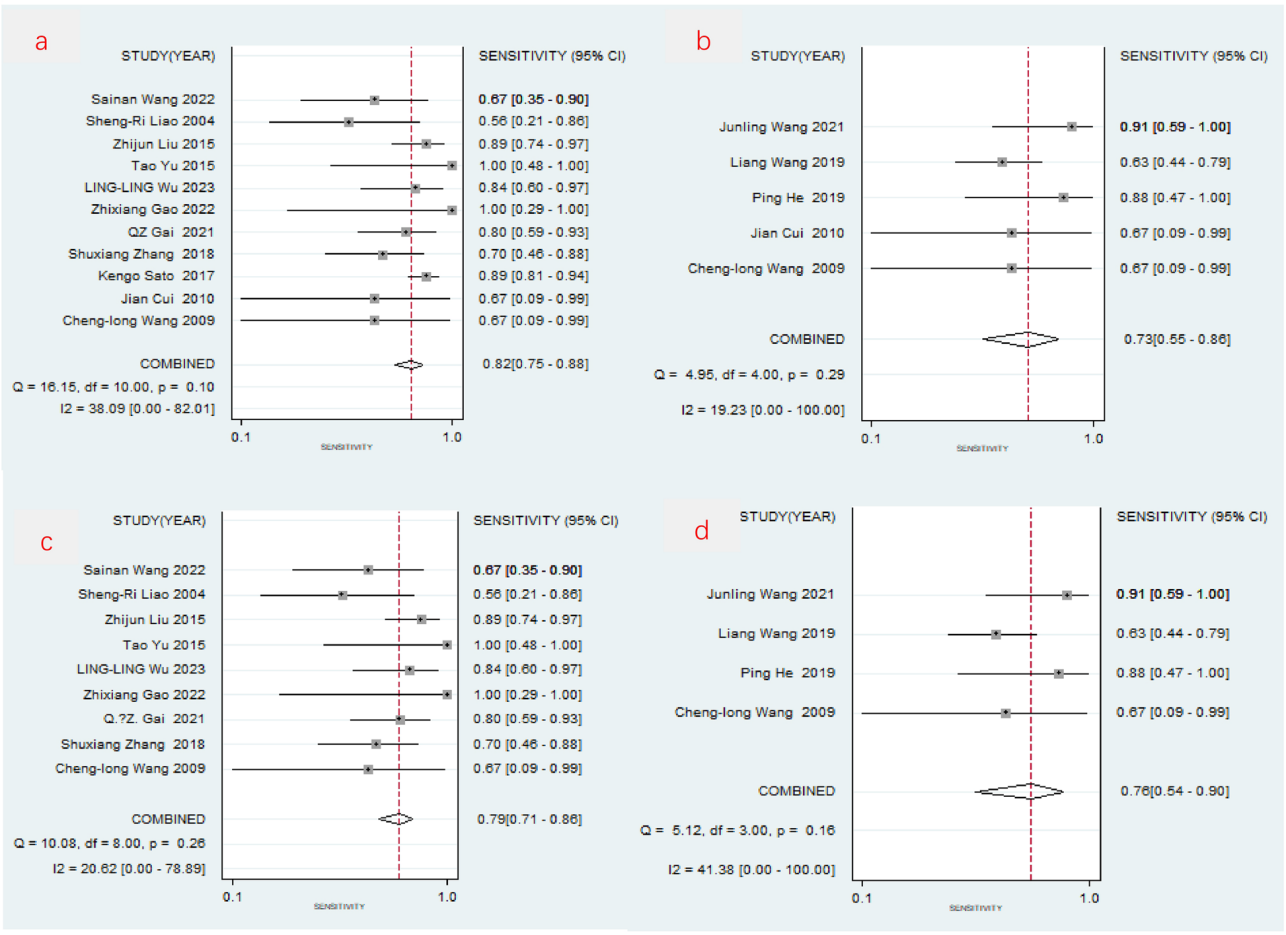
The combined sensitivity of O-CEGUS/D-CEGUS for discriminating ≤T1 and ≥T2 gastric cancer. **a** The sensitivity forest plot of the 11 included O-CEGUS–related papers. **b** The sensitivity forest plot of the five included D-CEGUS–related papers. **c** Sensitivity forest plots of nine O-CEGUS–related papers after excluding the sources of heterogeneity. **d** Sensitivity forest plots of four D-CEGUS–related papers after exclusion the sources of heterogeneity

**Fig. 4:**
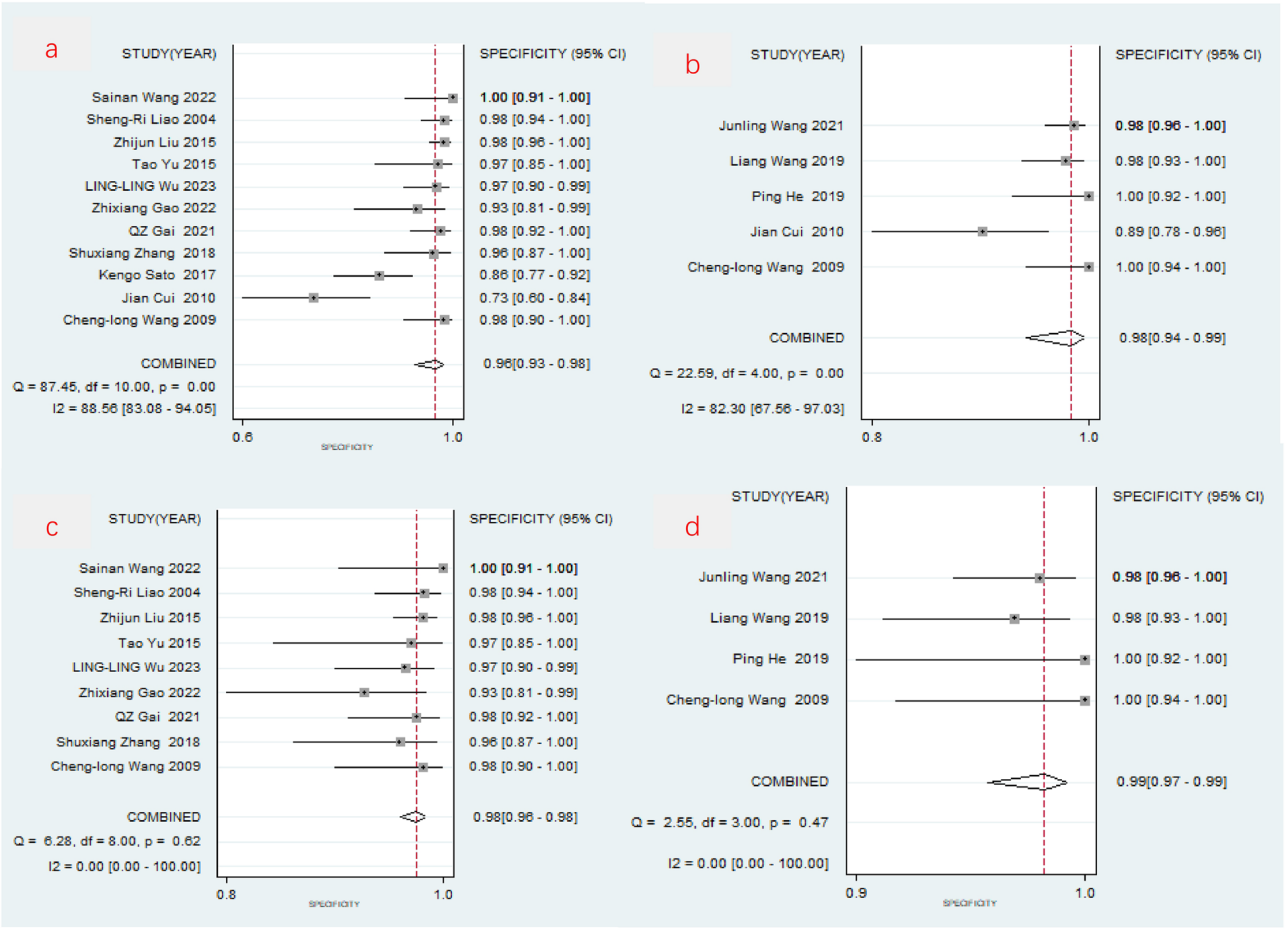
The combined specificity of O-CEGUS/D-CEGUS for discriminating ≤T1 and ≥T2 gastric cancer. **a** The specificity forest plot of the 11 included O-CEGUS-related papers. **b** The specificity forest plot of the five included D-CEGUS–related papers. **c** Specificity forest plots of nine O-CEGUS–related papers after excluding the sources of heterogeneity; Figure 4d: Specificity forest plots of four D-CEGUS–related papers after excluding the sources of heterogeneity

**Fig. 5:**
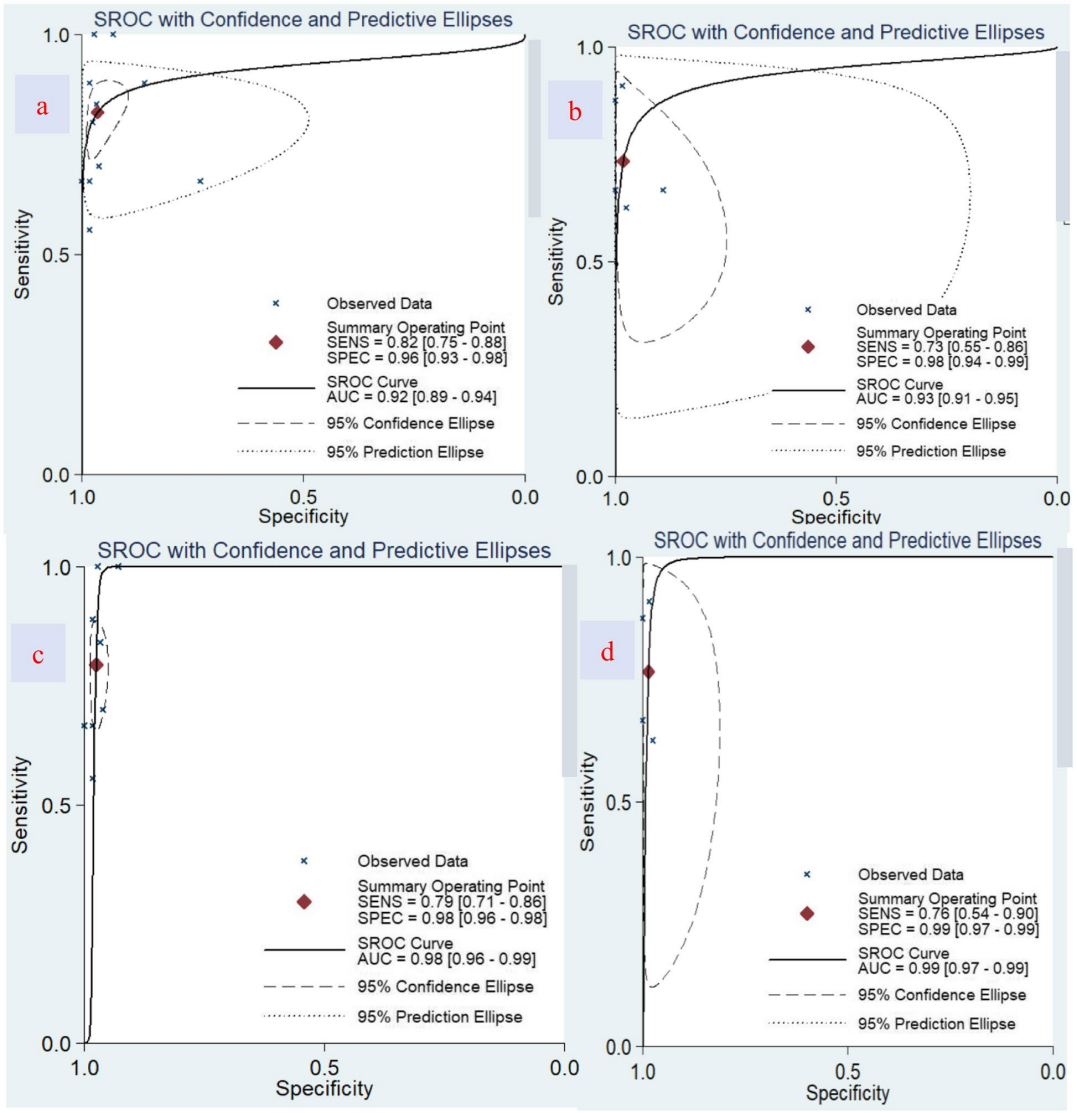
The sROC curves of O-CEGUS/D-CEGUS for discriminating ≤T1 and ≥T2 gastric cancer. **a** The sROC curves of the 11 included O-CEGUS–related papers. **b** The sROC curves of the five included D-CEGUS–related papers. **c** The sROC curves of nine O-CEGUS–related papers after excluding the sources of heterogeneity. **d** The sROC curves of four D-CEGUS–related papers after excluding the sources of heterogeneity. An empty circle represents each study’s sensitivity/specificity. A filled black circle represents the summary point for sensitivity/specificity. Dotted closed line, 95% confidence interval of the summary point; dashed closed line, 95% prediction region

**Fig. 6:**
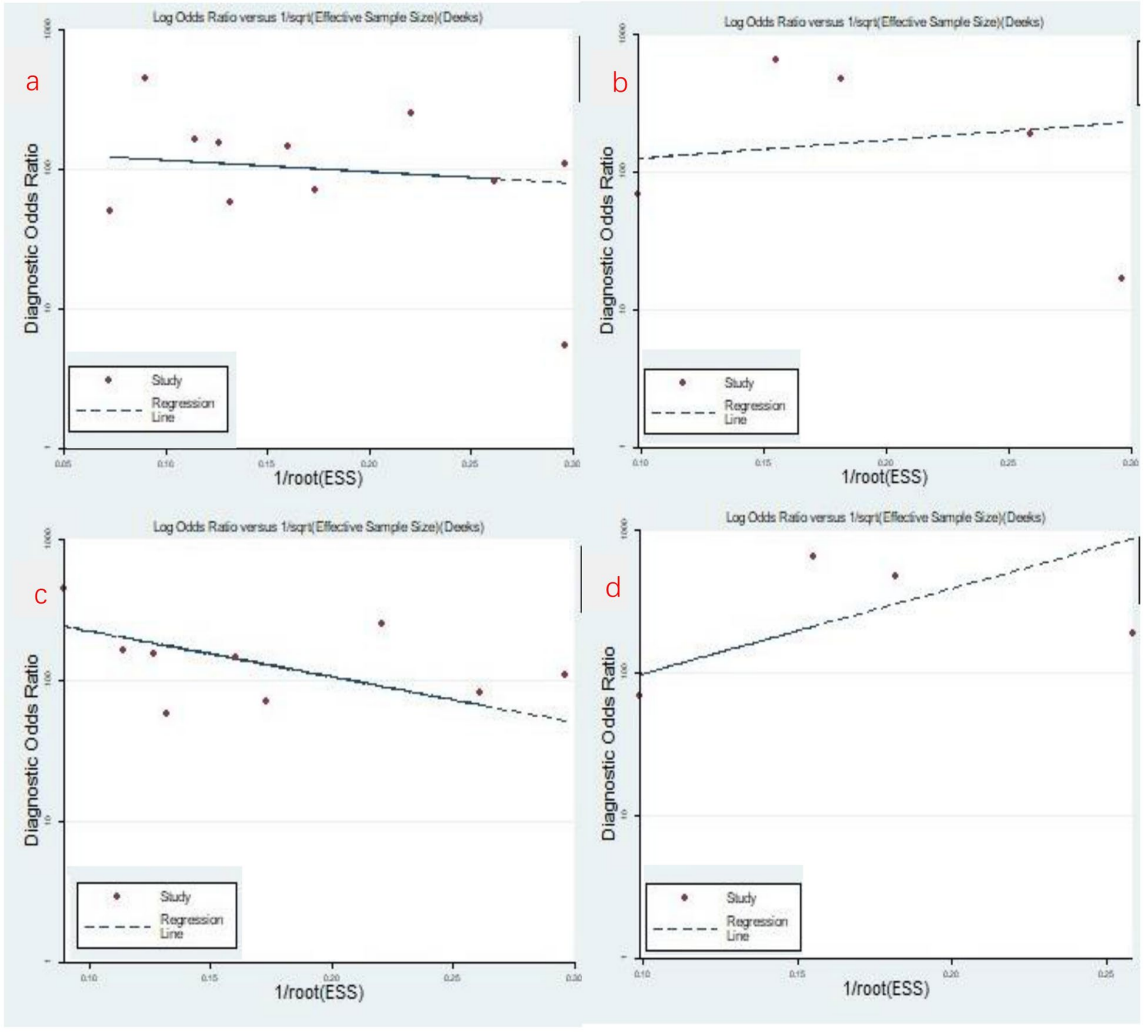
Funnel plot for publication bias among the included studies. **a** Funnel plot of 11 papers related to O-CEGUS. **b** Funnel plot of five papers related to D-CEGUS. **c** Funnel plot of nine papers related to O-CEGUS after excluding the sources of heterogeneity. **d** Funnel plot of four papers related to D-CEGUS after excluding the sources of heterogeneity. Each red circle represents a study

1. O-CEGUS: The combined sensitivity of the 11 O-CEGUS–related papers was 0.822 (95% CI = 0.753–0.875; Fig. 3a), the specificity was 0.964 (95% CI = 0.925–0.983), the PLR was 23.016 (95% CI = 11.199–47.302, Fig. 4a), the NLR was 0.184 (95% CI = 0.133–0.256), and the diagnostic odds ratio was 124.850 (95% CI = 62.039–251.250). The AUC was 0.92 (95% CI = 0.89–0.94), suggesting O-CEGUS had high accuracy in diagnosing gastric cancer (Fig. 5a). The forest plot revealed a sensitivity *I*^2^ of 38.09% (*P* = 0.10) and a specificity *I*^2^ of 88.56% (*P* < 0.01), suggesting significant heterogeneity regarding the specificity of the included literature, and further analysis of the sources of heterogeneity was needed.
2. D-CEGUS: The combined sensitivity (Fig. 3b) of the five D-CEGUS–related papers included in the study was 0.733 (95% CI = 0.550–0.860), the specificity (Fig. 4b) was 0.982 (95% CI = 0.936–0.995), the PLR was 40.349 (95% CI = 10.060–161.839), the NLR was 0.272 (95% CI = 0.149–0.497), and the diagnostic odds ratio was 148.286 (95% CI = 24.182–909.317). The AUC was 0.93 (95% CI = 0.91–0.95), suggesting that D-CEGUS had high accuracy in diagnosing gastric cancer (Fig. 5b). The forest plot revealed a sensitivity *I*^2^ of 19.23% (*P* = 0.29) and a specificity *I*^2^ of 82.30% (*P* < 0.01), suggesting significant heterogeneity in the specificity of the included literature, and further analysis of the sources of heterogeneity was needed.
3. Heterogeneous analysis: After excluding the studies by Cui et al. (17) and Kengo Sato et al. (20), which had the lowest specificities, heterogeneity was not noticed among the remaining nine studies on O-CEGUS (sensitivity: *I*^2^ = 20.62%, *P* = 0.26; specificity: *I*^2^ = 0.00%, *P* = 0.62) or the remaining four studies on D-CEGUS (sensitivity: I^2^ = 41.38%, *P* = 0.16; specificity: *I*^2^ = 0.00%, *P* = 0.47; Figs. 3c,d, 4c, d). The combined sensitivity and specificity of the nine O-CEGUS papers were 0.794 (95% CI = 0.710–0.859) and 0.976 (95% CI = 0.962–0.985), respectively, and the AUC was 0.98 (Fig. 5c). The combined sensitivity and specificity of the four D-CEGUS papers were 0.765 (95% CI = 0.543–0.899) and 0.986 (95% CI = 0.967–0.994), respectively, and the AUC was 0.99 (Fig. 5d). The indicators of the sensitivity forest plot are presented in Fig. 3, and the indicators of the specificity forest plot are presented in Fig. 4.
4. Publication bias: The literature included in this study was further examined for publication bias. Funnel plots, as depicted in Fig. 6a–d, were used to scrutinize the presence of publication bias among studies on O-CEGUS and D-CEGUS both before and after excluding sources of heterogeneity. The *P*-value derived from Deek’s test was 0.739 for the studies on O-CEGUS and 0.793 for the studies on D-CEGUS. The results suggest the absence of publication bias in this meta-analysis.
5. Sensitivity analysis: The stability and reliability of this meta-analysis were also evaluated by sensitivity analysis, in which one study was excluded at a time and the remaining data were combined to observe whether the heterogeneity and pooled effects changed. The results did not reveal any significant change in the heterogeneity and pooled effects of the meta-analysis in all cases, thereby indicating that this meta-analysis had high stability.

## Discussion

O-CEGUS and D-CEGUS have been feasibly used to diagnose gastric lesions, and their diagnostic value has gradually gained clinical recognition and attention [2]. However, their clinical efficacy in staging gastric cancer remains undefined. Our present study used meta-analysis to investigate the diagnostic efficacy of O-CEGUS and D-CEGUS in distinguishing stage ≤T1 and stage ≥T2 gastric cancer, and detailed analyses revealed that the AUCs for O-CEGUS and D-CEGUS were higher than 0.9 in the differential diagnosis of stage ≤T1 versus stage ≥T2 gastric cancer, thereby suggesting that CEGUS has diagnostic utility in staging early gastric cancer. We noticed the existence of high heterogeneity in the specificity of the included literature, and thus, the two papers with lowest specificities were excluded from the O-CEGUS and D-CEGUS forest plots, which subsequently eliminated the heterogeneity among the remaining papers. Consequently, nine studies related to O-CEGUS and four studies related to D-CEGUS were included in the next round of meta-analysis, which still gave AUCs > 0.9 for the efficacy of CEGUS in differentiating stage ≤T1 and stage ≥T2 gastric cancer. Thus, our meta-analysis proved both either O-CEGUS and D-CEGUS possessed high diagnostic efficacy in differentiating stage ≤T1 and stage ≥T2 gastric cancer.

Our present findings align closely with those of several previous studies conducted on similar grounds. Zhang et al. performed a meta-analysis involving seven studies, of which four were used for accuracy comparisons and two were used for sensitivity comparisons. The results revealed that O-CEGUS effectively differentiated early and advanced gastric cancer with AUC as high as 0.937 [28]. Similarly, Xu et al. conducted meta-analysis of six studies, finding that DCEUS exhibited an overall sensitivity and specificity of 0.94 and 0.91, respectively, in identifying T1–T2 and T3–T4 gastric cancer [29]. Furthermore, the meta-analysis by Zhang et al. incorporated eight studies on D-CEGUS, and its sensitivity and specificity for determining the T-stage of gastric cancer were 0.78 and 0.98, respectively for T1, 0.81 and 0.96, respectively, for T2, 0.88 and 0.85, respectively, for T3, and 0.81 and 0.96, respectively, for T4. The authors concluded that D-CEGUS had better accuracy than CT and EUS in differentiating T1–T2 and T3–T4 gastric cancer [30]. Lastly, the most recent meta-analysis by Nan et al. encompassed 21 studies on O-CEGUS extracted from various databases, and the study revealed AUCs of 0.93, 0.82, 0.87, and 0.97 for T1, T2, T3, and T4 gastric cancer, respectively [31].

However, our study differs significantly from the aforementioned research. Notably, the studies by Xu et al. [29], Zhang et al. [30], and Nan et al. [31] did not directly evaluate the diagnostic performance of CEGUS in distinguishing early gastric cancer from late-stage gastric cancer, which is crucial for guiding endoscopic intervention in patients with gastric cancer. The study by Zhang et al. [28] only analyzed the diagnostic performance of O-CEGUS in distinguishing early gastric cancer from late-stage gastric cancer without evaluating the clinical value of D-CEGUS in gastric cancer, and it included fewer O-CEGUS–related articles than the present study.

Taken together, there is sufficient evidence, along with outcomes, to recommend both O-CEGUS and D-CEGUS for everyday clinical practice in determining and differentiating the T-stage of gastric cancer, particularly in accurately discerning stage ≤T1 and stage ≥T2 gastric cancer before commencing endoscopic intervention.

Furthermore, our study both focused on the diagnostic efficacy of O-CEGUS/D-CEGUS for discriminating stage ≤T1 versus stage ≥T2 gastric cancer and analyzed O-CEGUS and D-CEGUS separately. Despite the smaller number of studies for D-CEGUS than for C-CEGUS, both methods exhibited the capability to differentiate between ≤T1 and ≥T2 gastric cancer. Consequently, these two different CEGUS methodologies represent viable alternatives aiding surgeons in selecting suitable treatment strategies. In addition, our findings suggest that although the diagnostic efficacy of D-CEGUS in differentiating ≤T1 and ≥T2 gastric cancer mirrors that of O-CEGUS, the former requires the administration of intravenous contrast agents such as Sonazoid® and SonoVue®. Given the potential risks of adverse reactions associated with Sonazoid® [32, 33] and SonoVue® [34, 35] alongside the higher examination costs of D-CEGUS, preliminary recommendations favor O-CEGUS for patients with gastric cancer undergoing ESD. This approach aims to streamline clinical decision-making, optimize patient outcomes, and maximize cost-effectiveness and convenience.

This study had several limitations. First, the limited number of studies on D-CEGUS in differentiating ≤T1 and ≥T2 gastric cancer might have hindered the comprehensive establishment of its efficacy and feasibility. Second, the predominantly Chinese origin of the included studies, with one article conducted in Japan, raises questions about the generalization of the conclusions regarding the application of CEGUS in gastric cancer staging. Third, the predominance of retrospective cohort studies among the included studied introduced the potential for recall and selection biases, necessitating further scrutiny of the accuracy of the data. Fourth, variation in ultrasound equipment across studies could contribute to diagnostic inaccuracy. Fifth, the use of different versions of TNM staging in the included studies might have limited the generalization and application of the findings. Lastly, although the diagnostic efficacy of EUS versus O-CEGUS/D-CEGUS for differentiating ≤T1 and ≥T2 gastric cancer is an area of interest, further validation through comparative trials is needed to draw definitive conclusions.

## Conclusion

Both O-CEGUS and D-CEGUS represent valuable tools for differentiating early-stage (≤T1) and advanced (≥T2) gastric cancer, thereby aiding in the selection of appropriate clinical treatment strategies for patients with very early gastric cancer. Given its simplicity and cost-effectiveness, O-CEGUS is likely to be favored as a staging method for gastric cancer prior to endoscopic interventions. The remarkable performance of CEGUS in gastric cancer staging underscores its significant clinical utility in evaluating gastric lesions, warranting further investigation and exploration into its diagnostic potential. The promising outcomes of CEGUS in this context highlights its potential to enhance patient care and treatment decision-making in the management of gastric lesions.

## Data Availability

The datasets used and/or analysed during the current study are available from the corresponding author on reasonable request.

## Abbreviations

CEGUS: Contrast-enhanced gastric ultrasonography
D-CEGUS: Double contrast-enhanced gastric ultrasonography
O-CEGUS: Oral contrast-enhanced gastric ultrasonography
sROC: Summary receiver operating characteristic
CI: Confidence interval
ESD: Endoscopic submucosal dissection
EUS: Endoscopic ultrasonography
CT: Computed tomography
PLR: Positive likelihood ratio
NLR: Negative likelihood ratio
AUC: Area under the curve
